# Development and validation of the SuPr-10 questionnaire for suicidality assessment in primary care patients with depressive symptoms

**DOI:** 10.1038/s41598-026-54258-w

**Published:** 2026-06-03

**Authors:** Carolin Haas, Philipp Sterner, Puya Younesi, Elena Wang, Gabriele Pitschel-Walz, Jochen Gensichen, Markus Bühner, Karoline Lukaschek

**Affiliations:** 1 Institute of General Practice and Family Medicine, University Hospital, LMU Munich, Munich, Germany; 2 Graduate Program “POKAL - Predictors and Outcomes in Primary Care Depression Care” (DFG-GrK 2621), Munich, Germany; 3 Institute of Psychology, University of the Bundeswehr Munich, Neubiberg, Germany; 4 Psychological Methods and Assessment, Department of Psychology, LMU Munich, Munich, Germany; 5 Department of Psychiatry and Psychotherapy, LMU University Hospital, Munich, Germany; 6 Department of Psychosomatic Medicine and Psychotherapy, University Hospital of Technical University of Munich, Munich, Germany; 7 Faculty of Medicine, Institute of Medical Information Processing, Biometry and Epidemiology (IBE), Chair of Public Health and Health Service Research, LMU Munich, Munich, Germany; 8 Information and Technology, TUM School of Computation, Technical University of Munich, Garching, Germany; 9 Institute of General Practice and Health Services Research, School of Medicine, Technical University Munich, Munich, Germany; 10 Division of Occupational Dermatology, Department of Dermatology, Ruprecht-Karls University Heidelberg, Heidelberg, Germany

**Keywords:** Diseases, Health care, Medical research, Psychology, Psychology

## Abstract

Suicidal ideation affects 10% of primary care patients. Although guidelines recommend screening, few tools are suitable for use in this setting, and protective factors are often overlooked. This study aimed to develop a brief questionnaire to assess suicidality in primary care. The Suicide Prevention for Primary Care Questionnaire (SuPr-10) was validated in 521 participants with a PHQ-9 score ≥ 6 across six subsamples. Its structure was analyzed using exploratory and confirmatory factor analysis, item analysis, and psychometric evaluation. Diagnostic accuracy was assessed via logistic regression and ROC analysis, and patient acceptance was measured in a follow-up survey. A two-factor structure with ten items was identified: I. protective scale (ω = .817) and II. risk scale (ω = .928), showing strong correlations with suicidality (r = –.529, .854) and depression (r = –.736, .626) scales. SuPr-10 showed acceptable discriminatory ability for identifying individuals with a history of suicide attempts (AUC = .765; 83% sensitivity, 56% specificity). It provides a structured assessment of suicidal tendencies and protective factors in primary care patients with depressive symptoms, supporting clinical decision-making and resource-focused communication. These findings are based on a clinically enriched, German-speaking sample, do not imply prospective prediction of suicidal behavior, and require validation in broader primary care populations.

**Supplementary Information:**

The online version contains supplementary material available at 10.1038/s41598-026-54258-w.

## Introduction

### Epidemiology of suicidality and definitions

Each year, more than 700,000 people die by suicide globally, making it one of the leading causes of death, accounting for 1.3% of all deaths worldwide^1^. A cross-national survey reported a 2.7% prevalence of suicide attempts^2^. In Germany, the suicide rate rose by 9.1% in 2022, from 11.1 to 12.1 per 100,000, placing the country in the lower mid-range within the European context^3^.

According to the World Health Organization (WHO), suicide is defined as “the act of deliberately killing oneself.” Suicidal ideation refers to “thoughts, ideas, or ruminations about the possibility of ending one’s life, ranging from a sense that one would be better off dead to the formulation of detailed plans” (ICD-10: R45.851^4^; ICD-11: MB26.A^5^). A suicide attempt is described as “a specific episode of self-harming behavior carried out with the conscious intention of ending one’s life” (ICD-10: T14.91^4^; ICD-11: MB23.R^5^).

### Primary care context: disclosure, screening, and secondary prevention

Older adults and individuals with multiple chronic conditions are particularly at risk for suicidal thoughts and behaviors^6,7^. Primary care settings include many older patients with chronic illnesses. Approximately 10% of general practice patients report current suicidal ideation^8^. Research indicates that such individuals frequently seek help from primary care physicians during suicidal crises^9–12^, although they rarely disclose suicidal thoughts unless directly asked^13,14^. Clinical guidelines therefore recommend low-threshold screening for suicidal ideation in patients with depression as part of secondary suicide prevention^15^.

### From risk-only screening to risk formulation

Contemporary models conceptualize suicidality as a dynamic, multi-dimensional process rather than a unitary construct. Frameworks such as the Integrated Motivational–Volitional (IMV) model^16,17^ and the Interpersonal Theory of Suicide^18^ emphasize the transition from psychological distress to suicidal ideation, and from ideation to action, as distinct but interacting processes. Accordingly, suicidality can be understood as a continuum ranging from passive death wishes and transient distress to active ideation, planning, and suicidal behavior. Differentiating these components is clinically important, as they reflect varying levels of risk and require different responses in practice. Though, substantial heterogeneity indicates that individuals may follow different pathways in the development of suicidal thoughts and behaviors^19^. Recent research highlights that the transition from suicidal ideation to behavior is not automatic but depends on additional factors such as capability, access to means, and contextual influences^20,21^. Similar perspectives further emphasize that suicidal behavior should be understood within a broader, person-centered and context-sensitive framework that integrates individual experiences with social and structural determinants^22^. Brief screening approaches in primary care can capture only selected aspects of the complex, multi-dimensional process of suicidality and are therefore primarily intended to support the identification of current risk indicators and to guide further clinical assessment, rather than to represent its full complexity. Accordingly, the present instrument is not designed to model suicidality in its entirety but to provide a brief, structured assessment of key elements along this continuum, with a particular focus on recent suicidal ideation, planning, and associated protective factors in a primary care context.

Positive Mental Health (PMH) moderates links between depression and suicidal ideation, and between ideation and attempts. Even with frequent lifetime ideation, individuals with high PMH show fewer lifetime suicide attempts^23–25^. PMH covers emotional, psychological, and social well-being. It integrates hedonic (positive affect, life satisfaction) and eudaimonic (growth, functioning) aspects^26,27^. PMH relates to greater life satisfaction, positive attitudes, and confidence, and correlates negatively with suicidal ideation and behavior^27^. Effective suicidality assessment extends beyond thoughts, plans, and attempts to include protective and buffering factors (e.g. positive mental health, life satisfaction, reasons for living, social support)^23–25,28^. Among depressed individuals, those without prior attempts endorsed greater family responsibility, greater social-disapproval concerns, stronger moral objections, better survival/coping skills, and greater fear of suicide than those with attempts^29^. Protective factors matter, but they do not cancel risk; they should not be used in a ‘risk − protection = suicide risk’ formula^30^. Protective factors add context, ease suicide-related conversations (especially in primary care), and indicate where to focus care (e.g., social support, self-efficacy).

According to NICE guidelines^31^, questionnaires are useful tools for exploration, follow-up, and documentation. While clinical judgment during debriefing remains essential, questionnaires can support risk assessment, particularly in risk formulation. This collaborative process between the patient and clinician identifies current risks and underlying causes, guiding treatment and safety planning. It considers historical and recent risk factors, as well as coping strengths and available resources^31^.

### Existing brief instruments in general practice

A systematic review^32^ of brief suicide risk tools suitable for general practice found that none assessed protective factors (including *RMTS-S*^33^*, SBQ-R*^33^*, K10*^34^*, SIS-Q*^35^*, SIDAS*^36^*, Gate question suicide attempt*^37^*, Gate question suicide ideation*^37^*, Feeling suicidal*^38^*, Wishing you were dead*^38^*, Thoughts of death*^38^*; PHQ-9 Item 9*^39^*)*. Moreover, no gold standard for suicidality assessment has yet been established^40^. The only instrument suitable for use in general practice that also includes protective elements is the P4—a four-item tool assessing past suicide attempts, suicide plans, the likelihood of suicide completion, and protective factors^41^. However, it has notable limitations. Its item wording (“hurt yourself”) is too vague and may include non-suicidal self-injury. Its dichotomous response format impedes the detection of subtle changes, and its internal consistency is low (Cronbach’s α = 0.44), limiting reliability. Additionally, it lacks data on diagnostic accuracy and was therefore excluded from the aforementioned review^32,]^^42^.

### Objectives

Building on the P4 and the PMH construct, this validation study aimed to develop and evaluate a psychometrically and factor-analytically optimized short questionnaire for assessing suicidality. We hypothesized that the questionnaire would exhibit a two-factor structure comprising a risk and a protective scale. We further expected the risk scale to show strong positive associations with established measures of suicidality and depression, and the protective scale to show inverse associations with these constructs, with overall stronger associations with suicidality than with general mental disorders. Finally, we anticipated that the questionnaire would provide added value over single-item screening approaches commonly used in primary care (particularly PHQ-9 item 9^43^) by capturing a broader and more differentiated profile of suicidality. Key innovations include the tool’s adaptation for primary care and the integration of protective factors. It was iteratively refined with input from clinicians and patients to enhance content validity and usability^44^.

## Methods

### Measures

#### The new questionnaire: suicide prevention in primary care “SuPr-X”

The preliminary version, SuPr-X, used "X" to denote the initially undetermined number of items, which was finalized through factor analysis during validation. Item selection for the preliminary version was guided by literature and current theoretical considerations relevant to primary care. The iterative development process and cognitive pretesting are described in detail elsewhere^45,46^. To ensure validity, cognitive pretesting with 10 general practitioners and 10 patients assessed comprehensibility, usability, and emotional burden, resulting in further refinements^47^: Key findings from interviews with GPs, patients, and a patient representative led to several adaptations of the questionnaire. GPs emphasized the need for time efficiency, so the number of items was reduced to the essential. Although we initially implemented skip logic to shorten completion time, we removed it at the GPs’ request because they were concerned about missing vulnerable individuals. The question order was revised to begin with positive mental health items (e.g., "self-confidence", “life goals”), followed by risk factors (suicidal tendencies), and ending with protective aspects, placing the more sensitive content in the middle. Layout and wording were revised to ensure clarity and ease of use. Patients often struggled with subtle distinctions, such as between "a desire to be dead" and "a wish to die", or "thinking about suicide" and "seriously considering suicide", while clearer differentiation was needed for reasons against suicide, like distinguishing between "responsibility for family" and "support from family". Ambiguous or redundant items, such as "Do you feel balanced?", were reworded or removed. Suicidal ideation items were softened (e.g., changing "Have you ever wished you were dead?" to "Have you ever wished you were not alive?") to reduce emotional burden. Patients generally preferred plain, direct language. After several evaluation rounds, most changes were completed early, and the cognitive pretest phase concluded with a stable version for the validation study. Additionally, the new instrument SuPr-X was evaluated during the validation study using an adapted acceptability questionnaire, based on the tool developed by Gräfe et al. for the PHQ-9 in 2004^48^.

The preliminary version comprises 17 items. It begins with four items on protective factors, adapted from the Positive Mental Health (PMH) Scale^27^—feeling confident, having life goals, feeling able to cope, and being satisfied—rated on a four-point Likert scale (0 = strongly disagree to 3 = strongly agree). This is followed by four dichotomous (yes/no) items assessing lifetime risk factors: previous psychiatric consultation, lifetime suicidal ideation, lifetime suicidal behavior (28% reattempt rate within 10 years^49^); and a family history of suicidality (associated with a fivefold increased risk^50^). Next, seven items assess suicidal ideation and behavior within the past two weeks, also rated on a four-point scale (0 = strongly disagree to 3 = strongly agree), in line with graded suicide risk models^51–53^. These cover: wish to die, suicidal thoughts, suicide method, suicide plan, urge to act on the plan, preparation for suicide, and self-harm/life-threatening behavior. The final two items ask about current suicide attempts and, if denied, explore preventive reasons. Prior factor analyses of other studies^28,54,55^ consistently identified six core protective motives: survival and coping beliefs, family responsibility, child-related concerns, fear of suicide, fear of social disapproval, and moral objections. Similarly, qualitative analysis of the P4’s preventive item revealed the "4 F’s": Family, Future Hope, Faith, and Fear of Failure^41,42^. Aligned with these findings and patient interviews, the new questionnaire provides multiple-choice options: *Confidence in your own strength, Faith or hope for improvement, Responsibility for others, Support from family, friends, or other social contacts, Fear of death or suicide, Moral or religious concerns, Concern about disapproval or others’ reactions, Other reasons (free text)*. This structure allows for a supportive opening and a resource-oriented conclusion by embedding questions on suicidal tendencies in between.

#### Additional measures

Participants also completed validated reference questionnaires with good psychometric properties to assess suicidality (Beck Scale for Suicide Ideation BSS^56^ & Brief Reasons for Living BRFL^54^), depression (Patient Health Questionnaire PHQ-9^43^), anxiety (Generalised Anxiety Disorder GAD-7^57^), post-traumatic stress disorder (Primary Care Post Traumatic Stress Disorder PC-PTSD-5^58^) and somatic symptoms (Patient Health Questionnaire PHQ-15^59^). For further details, see Supplement 1.

### Sample

A total of 521 participants were enrolled. To enhance ecological validity in primary care and to avoid missing suicidality outside formal major depression disorder (MDD), we required PHQ-9 ≥ 6 or endorsement of PHQ-9 item 9 (“Thoughts that you would be better off dead, or of hurting yourself”) irrespective of the total score (n = 4 with PHQ-9 < 6 but suicidal ideation). This approach allowed us to recruit patients with at least mild depressive symptoms without universally screening for suicidality. This threshold is also empirically anchored: in a PHQ-9 validation^48^, the mean for “any depressive disorder” was M = 11.7 (SD = 5.0), so M − 1 SD ≈ 6 captures the mild range. In a related POKAL-study in general practice, the mean PHQ-9 of all patients was ≈ 5.06 (95% CI 4.5–5.7)^60^, placing 6 at the upper confidence bound and aligning inclusion with routine primary-care distributions. Exclusion criteria included cognitive impairment, severe positive psychotic symptoms (e.g., hallucinations, disorganized thinking), and being underaged. Recruitment took place over 20 months (July 2022 to February 2024) using both traditional and web-based methods. Traditional recruitment occurred in 20 general practices, 12 psychotherapy practices, five inpatient psychiatric wards, and two day-clinics in Germany (primarily Bavaria) and Austria. The treatment settings were used for descriptive and comparative purposes only. Site characteristics (e.g., type of practice, urban vs. rural setting, and provider demographics) were not included as contextual variables in the statistical models or interpretation. Patients were approached by collaborating physicians or therapists, screened using the PHQ-9, informed about the study, and—if eligible—enrolled on-site after providing informed consent (n = 282). Web-based recruitment was carried out via social media and search engines (Facebook, Instagram, TikTok, Google Ads). Interested individuals completed a web-based prescreening using the PHQ-8 (a version of the PHQ-9 without the suicidality item). Those meeting the inclusion criteria (n = 1356) were invited to schedule an on-site appointment at the Institute of General Practice and Family Medicine at LMU Munich. Advertisements were geographically targeted to a 40 km radius around Munich to ensure feasibility of in-person attendance. During the on-site visit, study psychologists or physicians conducted an informational interview, confirmed eligibility, and provided the full questionnaire set for completion. Results were discussed in person, with special attention to responses indicating suicidal ideation. Participants recruited through this method (n = 239) were categorized by their main outpatient physician or therapist (e.g., GP, psychotherapist, psychiatrist). All participants received a €25 (US$ 26.96) shopping voucher as compensation. Detailed recruitment strategies and comparative analyses are reported elsewhere^61^.

For comparison of treatment settings, we divided the sample into six subgroups:

#### Outpatient/non-inpatient, n = 468:

1. General practitioner’s patients *“GP” n* = *211*
2. Psychotherapist’s patients *“PT” n* = *115*
3. Psychiatrist’s patients *“PSY” n* = *51*
4. Dayclinic patients *“DCP” n* = *74*

#### Inpatient, n = 53:

5. Non-acutely-suicidal inpatient participants *“NASIP” n* = *30*
6. Acutely suicidal inpatient participants *“ASIP” n* = *23.*

(These patients had attempted suicide within the past two weeks, were hospitalized under the Bavarian Psychiatric Care Act (BayPsychKHG) due to suicidal risk, or were temporarily restricted from leaving secure wards to prevent suicidal behaviour.)

### Statistical analysis

We used R 4.3.3.^62^. and SPSS version 29^63^ for all calculations.

#### Factor analysis

For the exploratory (EFA) and confirmatory (CFA) factor analyses, the dataset was randomly split into two independent subsets to examine items 1–11. Missing values were excluded listwise (n = 6). The adjusted EFA dataset included n = 256 complete cases, and the adjusted CFA dataset included n = 259. Sampling adequacy was supported by Bartlett’s test of sphericity, χ^2^(55) = 1512, *p* < 0.001; Kaiser–Meyer–Olkin (KMO) test (overall) = 0.865; and item-wise Measures of Sampling Adequacy (MSA) values ranging from 0.825 to 0.919, indicating that all variables were well suited for factor analysis. To determine the number of factors to retain, we applied parallel analysis, the Empirical Kaiser Criterion (EKC), and the Minimum Average Partial (MAP) test. We used Weighted Least Squares Mean and Variance adjusted (WLSMV) estimation for EFA and CFA, a robust method for ordinal data, and applied geomin rotation due to expected high factor correlations^64^. Global model fit was evaluated using the Root Mean Square Error of Approximation (RMSEA), Standardized Root Mean Square Residual (SRMR), and Comparative Fit Index (CFI), the Tucker–Lewis Index (TLI), and the normed chi-square (χ^2^ divided by degrees of freedom, χ^2^/df)^65^.

#### Item analyses

We analyzed communalities^66^, item difficulty and discrimination^67^, Spearman’s inter-item correlations^66,68^, internal consistency using McDonald’s omega^69^ and attenuation-corrected correlations with related constructs as an indicator for construct validity.

#### Diagnostic accuracy

As previous suicide attempts are a strong predictor of future attempts^70^, we used binomial logistic regression to assess how accurately the risk scale of the questionnaire (i.e., items assessing suicidal tendencies over the past two weeks) could predict a history of suicide attempts. We conducted a Receiver Operating Characteristic (ROC) analysis to evaluate sensitivity and specificity across various thresholds (n = 513, n = 8 missing). The Youden Index was used to identify the optimal cutoff, defined as the point maximizing the sum of sensitivity and specificity minus one. The positive predictive value (PPV) indicates the proportion of individuals classified as at risk by the SuPr-X risk scale who had actually attempted suicide in the past—thus representing a group with elevated suicide risk. Conversely, the negative predictive value (NPV) reflects the proportion of individuals not identified as previous attempters by the SuPr-X scale who indeed had no prior suicide attempt—indicating lower risk. Calculating PPV and NPV requires the a priori probability of the outcome, in addition to sensitivity and specificity. This a priori probability reflects an individual’s baseline risk, estimated based on both general prevalence and personal risk factors (e.g., mental health status).

#### Group differences

We conducted non-parametric Kruskal–Wallis tests to examine differences in suicidality and protective reasons across treatment groups and to explore response patterns among acutely suicidal patients. Effect sizes (r, according to Cohen) were interpreted as small (0.1–0.3), medium (0.3–0.5), and large (> 0.5)^68^.

### Ethical considerations

The authors affirm that all procedures contributing to this work comply with the ethical standards of the relevant national and institutional committees on human experimentation and with the Declaration of Helsinki (1975), as revised in 2013^71^. All procedures involving human subjects were approved by the medical ethics committee of LMU Munich on May 9, 2022 (project no. 22–0028). Written informed consent was obtained from all participants. Participants who were identified as suicidal were referred for outpatient psychiatric/psychotherapeutic care or, where indicated, for inpatient treatment. All participants were informed in the patient information leaflet that, in the event of acute suicidal ideation, they should immediately contact their health-care provider. In addition, all participants were provided with a printed list of local emergency contacts (e.g., psychiatric crisis service, on-call medical service, emergency number) and were advised to store these numbers on their phone. A customizable emergency-contact template to adapt to local services is available in Supplement 5. If, during recruitment/screening, sufficient self-protective capacity (i.e., the ability to maintain distance from suicidal impulses and to agree to a safety plan) could not be established, a study staff member personally accompanied the patient to the collaborating psychiatric inpatient unit (Department of Psychiatry and Psychotherapy at the University Hospital of LMU Munich) and handed them over to the on-call physician to discuss possible crisis admission.

## Results

Of the total sample, 67.8% were female (n = 353), 31.7% male (n = 165), and 0.38% non-binary (n = 2), with a mean age of 40.89 years (range: 18–83, SD = 14.32). The average PHQ-9 score was 14.8 (SD = 4.97; range: 5–27), indicating a moderate level of depressive symptom severity. No significant differences in sociodemographic characteristics or depression severity (as per inclusion criteria) were found between participants recruited directly by clinicians (traditional approach) and those recruited via web-based outreach (web-based approach, enrolled on site by study psychologists/physicians)^61^. Approximately half of the sample reported current suicidal tendencies (PHQ-9 item 9 > 0: 49.8%, n = 258; SuPr-X risk scale, items 5–11 > 0: 51.9%, n = 268), while 74.4% (n = 390) reported lifetime suicidal ideation (SuPr-X, item b). Additionally, 21.5% (n = 110) reported at least one previous suicide attempt.

In summary, the analyses suggest a two-factor structure reflecting risk and protective dimensions, with evidence of internal consistency, acceptable model fit, and associations in the expected directions with suicidality and depression. This structure appears clinically interpretable and promising, but should not be considered fully established and requires further validation:

### Structural validity

#### Exploratory factor analysis

Parallel analysis, Empirical Kaiser Criterion (EKC) and Minimum-Average-Partial-Test (MAP) suggested a two-factor solution explaining 61.62% (46.33% + 15.28%) of the variance:

I. *Protective scale* (referring to items about positive mental health in the last two weeks): Factor loadings item 1. 0.901; 2. 509; 3. 0.710; 4. 0.716
II. *Risk scale* (referring to items about suicidal tendencies of the last two weeks): Factor loadings item 5. 0.828; 6. 0.969; 7. 0.904; 8. 0.934, 9. 0.980; 10. 0.926, 11. 0.651).

The two-factor-solution resulted in high factor loadings with one slight double loading in item 10 (I. 0.307) and a factor correlation of −0.58 (Supplement 2, Table a).

#### Item analyses

Item difficulty for the protective factor scale was acceptable (0.32–0.47). For the risk scale, difficulty values ranged from 0.05 to 0.29, suggesting that certain aspects of suicidality may be extremely rare rather than poorly measured. Overall, item discrimination was good (0.45–0.86). Spearman inter-item correlations indicated strong internal consistency within each factor (Protective: r = 0.44–0.59; Risk: r = 0.36–0.81), with no signs of redundancy (all r < 0.90).

Communalities ranged from 0.35 to 0.81 (M = 0.55) for the protective scale, and from 0.42 to 0.96 (M = 0.81) for the risk scale, with an overall mean of 0.71—indicating high reliability.

### Item selection

#### Response pattern

**Fig. 1:**
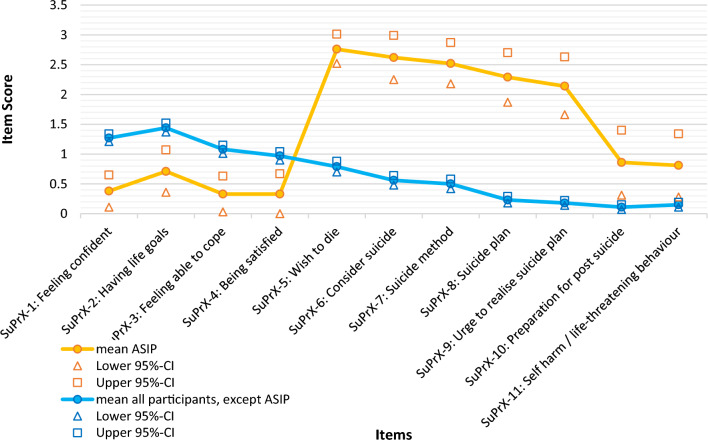
Response pattern of patients classified as acutely-suicidal (ASIP) vs. not.

Adapted from^46^, p.75.

In the absence of a gold standard reference^40^, it is essential to examine how response patterns of acutely suicidal individuals (ASIP) differ from those of other participants as an indicator of criterion validity. A comparison of average responses shows that individuals at acute suicide risk (n = 23) scored highest on items 5–9 (Fig. 1).

#### Statistical considerations

Items 10 (“In the last two weeks, I have made preparations for the time after my suicide.”) and 11 (“In the last two weeks, I have intentionally injured myself and/or put myself in danger, risking my own death.”) showed very low mean scores, high skewness, low difficulty, and weak item-total correlations. Their item discrimination was notably lower than that of the remaining risk items (Fig. 1). In the exploratory solution, Item 10 exhibited a double loading (I. 0.307 II. 0.926), and Item 11 showed below-average communality (m = 0.81 vs. item 11 = 0.42), indicating limited alignment with the target factors. The particularly low difficulty of these two items (item 10 = 0.05; item 11 = 0.06, m = 0.23) likely reflects low base rates of post-suicide precautions and self-endangering behaviors in the sample rather than an inherent measurement problem. Cronbach’s α improved slightly when these items were excluded, and model fit did not materially benefit from retaining them.

#### Conceptual considerations

The content of these two items may also be only partially specific to acute suicidality: “Preparation for post suicide” (Item 10) may index trait-like tendencies or life-circumstances rather than acute risk correlates (e.g., self-critical individuals being more likely to prevent discovery^72^; note-leaving as a marker for interpersonal conflict or loneliness^73–76^).

Item 11 may have been interpreted as non-suicidal self-injury (NSSI), a related but conceptually distinct phenomenon from suicidal intent^77^. Differentiating intentional self-harm without suicidal intent (NSSI) from suicidal behavior is crucial in clinical practice, as NSSI typically serves emotion-regulation or coping functions rather than reflecting a wish to die^78–81^. This distinction guides appropriate risk assessment, communication with the patient, and treatment planning. For clinicians interested in guidance on the assessment and management of NSSI in primary care, we refer readers to a comprehensive review summarizing epidemiology, assessment, and evidence-based interventions in this setting^82^.

#### Final selection

For reasons of parsimony and construct clarity, Items 10 and 11 were therefore removed. For detailed results, see Supplement 2, Table b. As a result of this item reduction, the final risk scale focuses primarily on recent suicidal ideation, planning, and behavioral impulses. This should be taken into account in clinical interpretation. The final questionnaire now comprises four items on the protective scale, five items on the risk scale, and one concluding item on protective reasons—resulting in a total of ten items, hence the final name: SuPr-10 (see Supplement 3).

A repeated EFA with the remaining items confirmed a two-factor structure, with high factor loadings and a factor correlation of –0.60 (see Supplement 2, Figure a).

#### Confirmatory factor analysis

Given the ordinal nature of the SuPr-10 risk-scale items, we report robust (WLSMV-based) fit indices. Conventional indices suggested good fit (χ^2^, p = 0.507; CFI = 0.988; TLI = 0.997; RMSEA = 0.055, 90% CI [0.028, 0.081]; **SRMR = 0.028]), but such indices can be optimistic under non-normal ordinal data. Robust indices indicated a mixed fit pattern (χ^2^(43) ≈ 68.49, p = 0.008; CFI = 0.954; TLI = 0.936; RMSEA = 0.144, 90% CI [0.082, 0.204]; **SRMR = 0.028]; χ^2^/df ≈ 1.59). While robust CFI/TLI and SRMR approach commonly used heuristics, the robust RMSEA was elevated. RMSEA represents residual misfit per degree of freedom and is sensitive to even minor local dependencies in the data. With ordinal indicators showing skewed and low-frequency responses on severe items, such residuals are common. Under WLSMV estimation of polychoric correlations, this can result in an apparently inflated RMSEA even when CFI, TLI, and SRMR indicate good fit. Taken together, the two-factor model can be regarded as acceptable though not optimal and was retained for theoretical interpretability. To maintain parsimony, we did not free additional parameters suggested by modification indices (e.g., cross-loadings or correlated residuals), as these changes would have yielded only trivial improvements in global fit and risked overfitting.

### Psychometric criteria and construct validity

McDonald’s omega indicated good internal consistency for both scales:

- I. Protective items 1–4: ω = 0.817.
- II. Risk items 5–9: ω = 0.928.

Table 1 presents construct validity, based on attenuation-corrected correlations with established measures of related and unrelated constructs. The final item of the SuPr-10, which assesses reasons for not attempting suicide, showed moderate to strong correlations with the corresponding factors from the BRFL scale (Spearman Rho correlation map: Brief Reasons For Living and Preventive Reasons SuPr-10., see Table 2).

**Table 1:** Attenuation-corrected correlations between SuPr-10 and other constructs.

	*SuPr-10* *protective scale* *Items 1.−4*	*SuPr-10* *risk scale* *Items 5.−9*		*Beck suicide scale BSS* *Item 1.−19*
*BSS*	−0.529**, n = 202	0.854**, n = 201		-
*Suicide attempt* *(SuPr-10 item c.)*	−0.131, n = 519	0.214**, n = 514		0.271**, n = 200
*PHQ-9*	−0.736**, n = 515	0.626**, n = 513		0.616**, n = 202
*GAD-7*	−0.416**, n = 509	0.179*, n = 507		0.183*, n = 200
*PHQ-15*	−0.264**, n = 467	0.205**, n = 465		0.178, n = 181
*PC-PTSD-5*	−0.113, n = 513	0.124, n = 511		0.061, n = 201
SuPr-10 (risk * protective)	−0.562**, n = 516			

Adapted from^46^, p.77; ** = p < 0.01; * = p < 0.05; BSS = Beck Suicide Scale, PHQ-9 = Patient Health Questionnaire 9 (depression), GAD-7 = Generalised Anxiety Disorder, PHQ-15 = Patient Health Questionnaire 15 (somatoform disorder), PC-PTSD-5 = Post Traumatic Stress Disorder 5.

**Table 2:** Spearman Rho correlation map: Brief Reasons For Living and Preventive Reasons SuPr-10.

***Preventive reasons (SuPr-10)***	Brief reasons for living (BRFL)					
	SC	RF	CC	FS	MO	SD
*Confidence in your own strength*	0.433**	0.074	−0.022	0.039	0.017	−0.044
N	477	493	191	499	497	500
*Faith or hope for improvement*	0.422**	0.199**	0.140	0.214**	0.130*	0.040
N	477	493	191	499	497	500
*Responsibility for others*	0.063	0.421**	0.343**	−0.012	0.347**	0.042
N	477	493	191	499	497	500
*Support from family, friends*	0.183**	0.294**	0.085	0.122*	0.076	0.110*
N	477	493	191	499	497	500
*The fear of death or suicide*	0.039	0.000	−0.078	0.471**	0.057	0.151**
N	476	492	190	498	496	499
*Moral or religious concerns*	0.046	0.077	0.041	0.051	0.450**	0.106
N	477	493	191	499	497	500
*Concern about disapproval*	−0.033	0.041	−0.020	0.044	0.106*	0.295**
N	477	493	191	499	497	500

Adapted from^46^, p.76; ** = p < 0.001; * = p < 0.05; SC = Survival and coping beliefs (item 3 + 12); RF = Responsibility to family (item 2 + 5); CC = Child concerns (item 4 + 7); FS = Fear of suicide (item 1 + 10); MO = Moral objections (item 6 + 9); SD = Fear of social disapproval (item 8 + 11).

### Criterion-related evidence using proxy outcomes

The SuPr-10 risk scale (items 5–9, assessing suicidal tendencies) significantly predicted previous suicide attempts (p < 0.001)—an important indicator of future risk. Each one-point increase in the SuPr-10 score was associated with a 1.25-fold increase in the odds of a prior attempt (Exp(B) = 1.250, 95% CI [1.183, 1.320]). ROC analysis yielded an AUC of 0.765 (95% CI [0.712–0.819]), indicating acceptable discriminatory ability of the SuPr-10 risk scale to distinguish between individuals with and without a history of suicide attempts. As previous suicide attempts are a well-established proxy indicator of elevated risk^83^, these findings may be considered a clinically promising discrimination. However, they do not allow conclusions regarding prospective prediction of future suicidal behavior. By comparison, the AUC values for the BSS (0.652, 95% CI [0.574–0.730]) and the PHQ-9 (0.696, 95% CI [0.639–0.753]) were lower, suggesting that SuPr-10 may provide a more differentiated assessment in this context, although differences in discriminatory performance should be interpreted cautiously.

#### Positive and negative predictive value

Since universal screening is not intended, the calculation of the positive predictive value (PPV) and negative predictive value (NPV) was based on the a priori probability for individuals with depressive symptoms, rather than the general population prevalence of suicide attempts. In the total sample (n = 511; 10 missing), the prevalence of lifetime suicide attempts was 21.5% (n = 110), reflecting the elevated risk associated with depressive symptoms (Table 3).

**Table 3:** Diagnostic accuracy of SuPr-10 risk scale (Item 5.−9).

Cut-Off	Sensitivity [95% CI]	Specificity [95% CI]	Youden- Index	PPV depressive symptoms*	NPV depressive symptoms*
≥ *1*	83.17% [74.7–89.2]	56.17% [51.4–60.9]	39.34%	34.20%	92.42%
≥ *2*	78.22% [69.2–85.2]	68.77% [64.1–73.0]	46.98%	40.68%	92.02%
≥ *4*	59.41% [49.7–68.5]	79.90% [75.8–83.5]	39.31%	44.74%	87.79%
≥ *7*	38.61% [29.7–48.4]	89.59% [86.3–92.2]	28.20%	50.39%	84.20%

Adapted from^46^, p.80; A-priori probability suicide attempt: 21.5% in study sample with depressive symptoms. Full list, see Supplement 2, Table c.

### Preliminary clinical thresholds

**Fig. 2:**
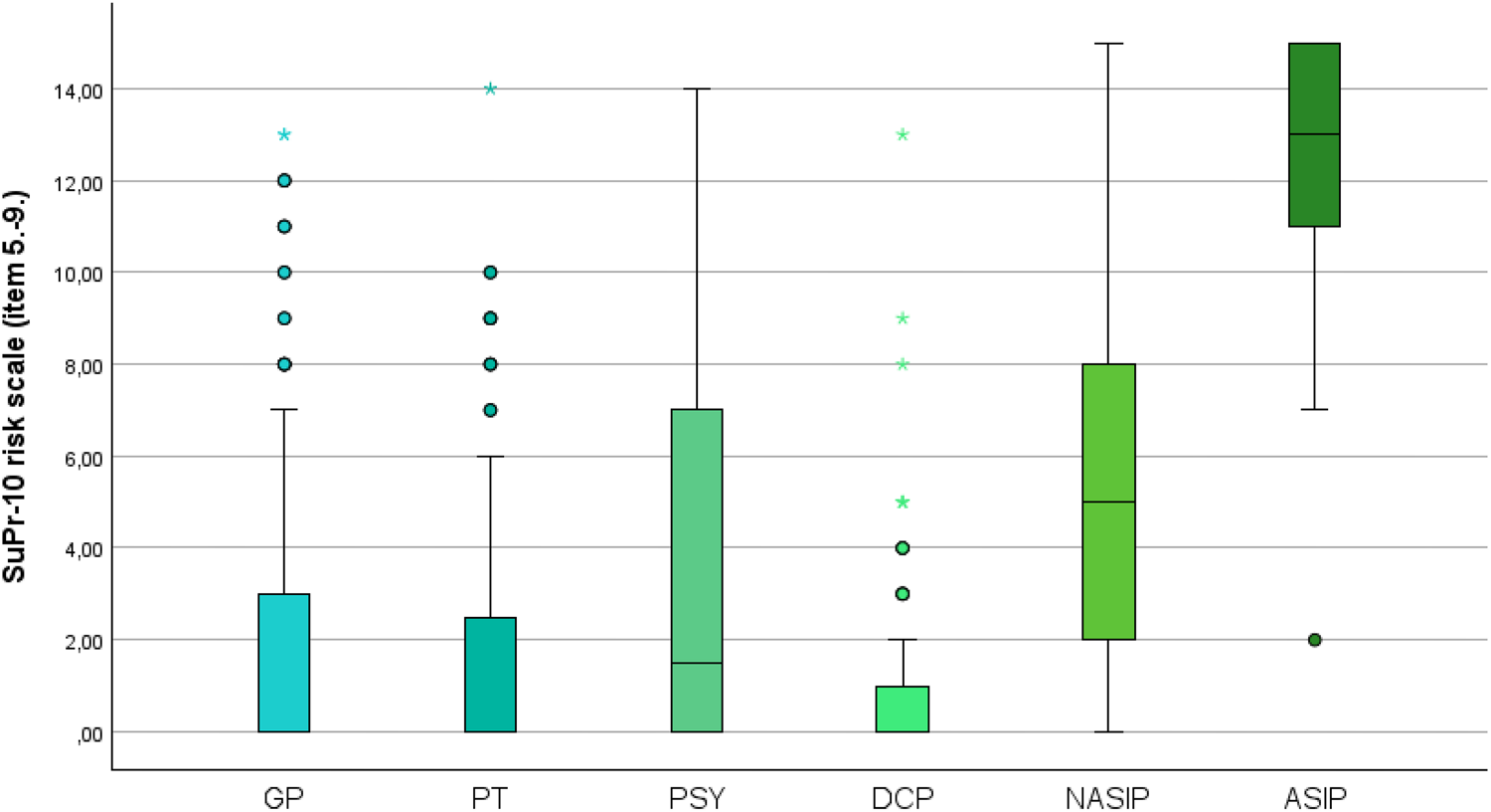
Distribution of SuPr-10 risk scale (remaining items 5.−9.), Group Comparison.

Adapted from^46^, p.79; GP = General practitioner’s patients, PT = Psychotherapist’s patients, PSY = Psychiatrist’s patients, DCP = Day clinic patients, NASIP = Non-acutely suicidal inpatient participants, ASIP = Acutely suicidal inpatient participants.

In terms of criterion validity, the response patterns of acutely suicidal patients (ASIP, n = 23) should inform threshold recommendations. All ASIP patients identified by clinicians in protected psychiatric wards scored above 7 on the SuPr-10 risk scale (items 5–9), with one exception (*outlier, see*. Fig. 2) Their mean score was 12.33 (95% CI: 10.80–13.87). In contrast, the non-acutely suicidal inpatient group (NASIP) had a mean score of 5.57 (95% CI: 3.86–7.27). ASIP participants scored significantly higher than all other groups on the SuPr-10 risk scale (non-inpatients: p < 0.001, r = 0.332–0.366, moderate effect sizes; NASIP: p = 0.036, r = 0.163, small effect size). On the SuPr-10 protective scale, ASIP participants never exceeded a score of 4, with a mean of 1.76 (95% CI: 1.00–2.52). Notably, a score of 4 also marked the upper limit of the interquartile range in the NASIP group. Sensitivity and specificity for predicting a history of suicide attempts were low, with the highest combined accuracy (Youden index) at a threshold of ≤ 4. For identifying current suicidal tendencies (SuPr-10 risk scale > 0), the best Youden index was achieved at a threshold of ≤ 5.

### Protective reasons

The social dimension emerged as the most relevant protective factor: 59.2% of participants cited responsibility for others, and 54.1% mentioned social support as a reason for not attempting suicide. Among inpatients, the importance of social support was even higher (ASIP: 63.2%; NASIP: 65.5%).

Internal motives—such as confidence in overcoming the crisis, inner strength, and hope—also played a significant protective role but were reported less frequently in more intensive treatment settings. Fear of death or suicide was mentioned by 32.9% of participants, while moral concerns (13.1%) and fear of social disapproval (15.4%) were less common.

These reasons were categorized as internal (e.g., confidence in one’s own strength, belief in recovery, personal responsibility for others) versus external (e.g., support from others, fear of death, religious or moral concerns, fear of others’ reactions).

The acutely suicidal group (ASIP) was significantly less likely to report internal reasons compared to all non-inpatient groups (p < 0.001), with small effect sizes (r = 0.158–0.186):

- H(5) = 20.997, p < 0.001.
- GP: z = 4.123, p = 0.001, r = 0.186.
- PT: z = 3.796, p = 0.002, r = 0.171.
- PSY: z = 3.796, p = 0.007, r = 0.158.
- DCP: z = 4.038, p = 0.001, r = 0.182.
- NASIP: z = 2.062, p = 0.588.

For a graphical overview, see Supplement 4.

### Acceptance of new instrument

The vast majority of participants (94.6%, n = 434/459) found the questionnaire acceptable, with 89.2% (n = 411/461) reporting it was not difficult to complete and 98.3% (n = 450/458) saying it was not too time-consuming. Regarding its relevance for medical care, 88.8% (n = 388/438) believed the questionnaire could contribute to improved treatment, and 96.5% (n = 442/458) stated they would be willing to complete it again in the future. Among practitioners, 96.2% (n = 25/26) rated the questionnaire as useful; 89.3% (n = 25/28) would use it in their routine care [60.7% (n = 17/28) for selected patients—especially those with depression—and 28.6% (n = 8/28) for all patients with mental illness].

## Discussion

The objective of this study was to develop a suicidality questionnaire that explicitly includes protective factors—an element largely underrepresented in existing instruments—while ensuring adequate psychometric quality through careful design. The study evaluated the questionnaire’s construct validity and its ability to predict previous suicide attempts as an indicator of its practical applicability.

### Construct validity

The significant negative correlations between the SuPr-10 protective scale and established measures of suicidality and various mental disorders support its convergent validity in capturing factors that reduce suicide risk. Conversely, the risk scale showed significant positive correlations with the same measures, further supporting its convergent validity (Table 1). In addition, the final item assessing reasons for not attempting suicide demonstrated convergent validity through moderately strong correlations with the BRFL subscales, confirming the relevance of the modified selection and adjustments (Table 2).

### Cut-off considerations

According to the Youden Index^84^, a cut-off of ≥ 2 offers the highest combined diagnostic accuracy for the SuPr-10 risk scale. However, the Youden Index does not account for the unequal clinical consequences of low sensitivity versus low specificity. In suicide risk screening, insufficient sensitivity carries greater clinical risk. Therefore, we recommend a lower cut-off to maximize sensitivity while maintaining acceptable overall accuracy. Runeson et al. (2017) proposed pragmatic thresholds of > 80% sensitivity and > 50% specificity as minimum standards for suicide screening tools^85^. Based on these criteria, there is strong justification for setting an initial cut-off at > 0 on the SuPr-10 risk scale (suicidal tendencies subscale), which yields a sensitivity of 83% and specificity of 56%, thus meeting both thresholds.

#### Application of the SuPr-10 risk scale in primary outpatient care

The cut-off values were established from in-sample ROC evaluation, and we did not conduct internal resampling (cross-validation or bootstrapping). As a result, performance estimates may be optimistically biased and sensitive to the case mix and prevalence in this sample. The proposed cut-off values should be understood as preliminary decision-support anchors derived from in-sample analyses. They require replication, cross-validation, and calibration in independent samples before being considered for routine clinical implementation. In routine practice, the SuPr-10 may be administered as an adjunct whenever clinicians suspect at least mild depressive symptomatology, preferably in addition to the PHQ-9, and the results discussed collaboratively with the patient to support shared understanding and safety planning. Although the response patterns of the acutely suicidal inpatient group provide clinically meaningful reference points, they should be interpreted with caution, given the limited sample size. Threshold recommendations should therefore be regarded as provisional and require validation in larger and independent samples. Our threshold recommendations can offer initial guidance for use in primary care and future research:

- An increased risk for suicidal behavior should be assumed if a patient scores > 0 on the SuPr-10 risk scale (sum of items 5–9 assessing suicidal tendencies). In such cases, general practitioners should be particularly attentive and initiate safety planning^86^. The protective reasons assessed in the final item of the SuPr-10 can directly support this process (for practical guidance, see e.g^87^.). Initial interventions should focus on strengthening self-efficacy and mobilizing social support.
- If a patient scores ≥ 4 points, inpatient treatment should be considered and discussed with the patient.
- A score of ≥ 7 indicates a response pattern comparable to that of acutely suicidal patients in secure psychiatric wards. In such cases, close monitoring is essential and is most reliably ensured in an inpatient setting.
- Caveat: Even a score of zero on the SuPr-10 risk scale does not rule out suicidal behavior^88,89^.

Recommended same-visit procedures include means-restriction counseling, a written safety plan, and provision of local crisis contacts, followed by timely re-assessment (e.g., within days for elevated risk). We supply editable handouts (Supplement 5) with an application guide, crisis-management tips, a patient safety-plan worksheet, and a regionalizable emergency contact list to facilitate implementation. The German-language versions of the handouts are provided in the appendices of the author’s published dissertation^46^.

#### Additional implications for SuPr-10 protective scale

In addition to the SuPr-10 risk scale, the protective scale offers contextual information on patients’ current emotional state and available resources. However, we want to emphasize that the protective scale should not be interpreted as an indicator of suicide risk on its own. Its primary function is to contextualize risk and to support clinical exploration, rather than to discriminate between levels of suicide risk. Consistent with this, the protective scale correlates more strongly with depression than with suicidality (Table 1). Therefore, a low protective score *in isolation* does not indicate high suicide risk. Clinical decisions should be guided primarily by the risk scale and clinical judgement; the protective scale is best used to contextualize risk (e.g., identify low internal/external resources) and to guide intervention focus (e.g., strengthening self-efficacy, mobilizing social support). If all risk items are negated but the protective score is very low, regular reassessment is advised. Importantly, the protective scale allows for a gentler entry into suicide-related conversations—an approach especially valued by general practitioners. Practice guidance for outpatient care (interpretive, non-diagnostic indicators):

- Protective ≤ 5: associated with higher levels of suicidality in this sample; consider closer follow-up. However, this threshold should be interpreted cautiously and not as a stand-alone indicator of suicide risk.
- Protective ≤ 4: shows the best accuracy for identifying individuals with a history of suicide attempts and may indicate increased risk of current depression and suicidality; review suicide- and psychiatric history, identify and mobilize social supports, assess coping strategies and help-seeking behavior.
- Protective ≤ 2: aligns with the response pattern of the acutely suicidal subgroup in secure psychiatric wards; intensify monitoring; ensure rapid reassessment.
- Clinical reminder: Lower scores on the protective scale may be associated with increased vulnerability and reduced internal and external resources, but should not be interpreted as indicators of suicide risk in isolation. Likewise, a high protective score does not rule out suicidal behavior^88^. Clinical decisions should be guided by the risk scale in conjunction with clinical judgment. The protective profile may help to contextualize risk and to identify potential targets for intervention (e.g., strengthening self-efficacy or mobilizing social support).

#### Potential use of protective scale as prescreening

If the protective scale had been used as a prescreening tool, with the risk scale administered only when patients scored ≤ 5, ten individuals at increased risk (≥ 4 on the risk scale) would have been missed in the GP setting. Therefore, we recommend administering the full questionnaire—including both protective and risk factors—to ensure comprehensive assessment. Importantly, asking about suicidality does not increase suicidal thoughts or behavior^90^. Beyond these methodological considerations, the practical relevance of the instrument in clinical settings warrants further discussion.

### Acceptance and clinical implications

Beyond its psychometric properties, the instrument captures clinically meaningful aspects of patients’ current experience. The risk scale reflects recent suicidal thoughts, planning, and behavioral impulses, while the protective scale provides insight into perceived coping capacity, emotional stability, and available internal and external resources (such as social support, responsibility for others, or moral and religious concerns). Rather than serving as a deterministic indicator of suicide risk, the questionnaire is intended to support structured clinical understanding and facilitate discussion of the patient’s current situation. In this sense, the questionnaire is not intended as a stand-alone measurement tool, but as a structured conversation aid that helps clinicians ask about suicidality in a direct and non-stigmatizing way. By linking assessment with the identification of protective resources, it supports the integration of assessment and intervention and provides concrete starting points for collaborative safety planning. This may be particularly relevant in primary care, where clinicians may benefit from structured guidance in addressing suicidality openly and confidently. This perspective aligns with recent work emphasizing that suicidal behavior should be understood within a broader, person-centered and context-sensitive framework that integrates individual experiences with social and structural determinants^22^.

Our findings indicate good acceptability and feasibility of the SuPr-10—comparable to the acceptance of depression screening with the PHQ-9^48^—but do not constitute evidence of clinical effectiveness or impact on patient outcomes. Other studies also confirm that patients generally support the assessment of suicidality in primary care settings^91^. While acceptability and perceived usefulness are important prerequisites for implementation, further research is needed to evaluate the impact of the SuPr-10 on clinical processes and patient outcomes.

#### Advantages of using SuPr-10 as a complement to PHQ-9 over PHQ-9 alone

As a complement to the PHQ-9, the SuPr-10 allows for a more nuanced assessment of suicidality, addressing the limitations of the PHQ-9’s single suicidality item. Single-item measures are insufficient for capturing the complexity of suicidality and remain controversial^32,37,92,93^. If suicidality had only been explored in patients who endorsed the PHQ-9 suicidality item, 7% (n = 36/521) of individuals reporting suicidal tendencies in the past two weeks would have been missed. Notably, 19% (7/36) of these missed cases scored ≥ 4 on the SuPr-10 risk scale, a clinical threshold at which inpatient evaluation should be considered. These findings underline the added clinical utility of administering the SuPr-10 alongside the PHQ-9 to reduce false negatives and to trigger safety planning in patients who might otherwise be overlooked. When used alongside the PHQ-9, the SuPr-10 offers a more comprehensive suicidality assessment by incorporating both risk factors and protective elements. In more intensive treatment settings, patients reported lower levels of self-efficacy and relied more heavily on external protective factors—with social support emerging as the most relevant across all groups. These findings can assist general practitioners in risk assessment and safety planning, with an emphasis on strengthening self-efficacy and mobilizing social resources.

### Suicide prediction vs. suicide prevention

Suicide prediction remains difficult. Over the past 50 years, predictive models have performed only slightly better than chance^88^. The SuPr-10 does not provide a reliable diagnostic in the classical sense, but rather serves as an assessment tool, to support structured exploration and communication of suicidal experiences within the clinical encounter and to support collaborative risk formulation and clinical decision-making in routine care.

Awareness of the proposed thresholds can support treatment planning and thus contribute meaningfully to suicide prevention. However, it is crucial to emphasize that the use of such a questionnaire must not lead to a false sense of security. Ultimately, clinical judgment remains the most important factor in decision-making.

### Strengths and limitations

#### Development and content validity

As part of the Research Training Group POKAL, the development of the SuPr-10 benefited from extensive interdisciplinary expertise. The instrument is grounded in a solid theoretical framework and was iteratively refined through practical application and feedback, incorporating the perspectives of general practitioners and patients. This process has resulted in high content validity and usability.

#### Model fit and estimation considerations

The overall model fit can be considered acceptable but not optimal. While robust CFI, TLI, and SRMR indicated acceptable fit, the robust RMSEA and significant chi-square suggested some global misfit. The threshold recommendations for RMSEA predominantly stem from research focused on Maximum Likelihood (ML) estimation^65^. As our analysis employs the Weighted Least Squares Mean and Variance adjusted (WLSMV) estimation method, these thresholds may not be directly applicable^94^. Though, this pattern is not unusual in models estimated with WLSMV using ordinal and skewed indicators, where small local dependencies or sparse response categories can inflate global misfit indices such as RMSEA, even when other indices are satisfactory^95^. RMSEA reflects average residual misfit per degree of freedom and therefore tends to penalize minor discrepancies across all items rather than indicating a single problematic parameter. With 43 degrees of freedom and a moderate sample size (N = 521), the elevated RMSEA likely reflects these data characteristics rather than substantive model misspecification. The two-factor structure was retained for its theoretical coherence and parsimony, as adding parameters suggested by modification indices would have yielded only trivial improvements in fit while reducing interpretability. Considering the consistent pattern of the remaining robust indices (CFI, TLI, SRMR) and the acceptable χ^2^/df ratio, the model can be regarded as psychometrically promising, though further work—including measurement-invariance testing and external cross-validation—is warranted to confirm the stability of the structure.

#### Sampling frame and PHQ-9 selected cohort

Lowering the PHQ-9 cut-off from the commonly used ≥ 10 to ≥ 6 enabled the inclusion of 43 patients with a history of suicidal ideation and 8 with current suicidal ideation who would have been missed by standard PHQ-9 prescreening. Since many PHQ-9 items reflect symptoms found in other disorders, the sample also included patients with anxiety, trauma-related, and psychosomatic complaints. While broader use is conceivable, the current recommendations are based on a PHQ-9-selected sample. Further validation is needed before generalizing to other diagnostic groups.

#### Generalizability across languages and settings

The current recommendations primarily apply to adult patients with at least mild depressive symptoms, as defined by the PHQ-9 inclusion criteria used in this study. The sample represents a clinically enriched population rather than an unselected primary care population. Caution is therefore warranted when generalizing these findings beyond similar patient groups, as prevalence, case-mix, and response patterns may differ in broader primary care settings. Moreover, validation was restricted to German-speakers. Broader use will require cross-cultural validation, including careful translation, testing in target populations, and calibration of cut-offs across settings.

#### Criterion validity proxy and ASIP comparator

In the absence of a gold standard for criterion validity, 23 acutely suicidal patients were included for comparison of response patterns. While this is a relatively small sample for making representative claims, most suicidality questionnaires are validated only in terms of construct validity—making this a notable strength of the study.

#### Lack of prospective outcomes and predictive validity

We collected no prospective follow-up data, and no prospective validation is currently planned within this project; therefore, we cannot draw conclusions about the predictive validity of SuPr-10 scores for subsequent suicidal behavior. It remains unclear whether, and to what extent, participants exhibited suicidal behavior after assessment. The SuPr-10 is, however, in use within an ongoing depression intervention study in the POKAL consortium (expected to run until 2027), which may provide a foundation for secondary longitudinal analyses in future work. As with all screening instruments for suicidality, predictive power remains highly limited.

### Conclusion

The SuPr-10 showed acceptable psychometric performance, evidence of content validity, and high acceptability among patients with depressive symptoms in this sample. It offers a brief, structured assessment of both risk and protective factors for outpatient and primary care, while acknowledging the inherent limits of predicting suicidal behavior. As a complement to the PHQ-9, the SuPr-10 can provide a more nuanced view of suicidality. Patient responses should always be reviewed and discussed directly with the patient to ensure that any concerns are addressed promptly. Treatment decisions should not rely on questionnaire results alone. Used alongside the PHQ-9 and clinical judgment, the SuPr-10 may support screening (risk scale to flag current suicidality) and clinical decision-making (protective scale to contextualize risk and guide safety planning). In short, the SuPr-10 is best understood as a tool to support structured clinical dialogue and risk formulation, rather than as a stand-alone predictive instrument. External and cross-cultural validation will be essential before broader application, and the proposed cut-offs should be considered preliminary until confirmed in independent samples.

## Supplementary Information

Supplementary Information.

## Data Availability

Data, analytic code, and research material supporting this study’s findings are available from the DFG-GrK 2621/POKAL studies but are not publicly accessible due to licensing restrictions. They can be obtained upon reasonable request by contacting “Stiftung Allgemeinmedizin – The Primary Health Care Foundation” at www.stiftung-allgemeinmedizin.de or via email at [office@stiftung-allgemeinmedizin.de](mailto:office@stiftung-allgemeinmedizin.de).
