# Supplementary material for: Development and validation of the SuPr-10 questionnaire for suicidality assessment in primary care patients with depressive symptoms
Source: Sci Rep. 2026 Jun 3;16:17127. doi: 10.1038/s41598-026-54258-w (PMC13234288; doi:10.1038/s41598-026-54258-w)
Supplement: Supplementary file 1 — Supplementary Information. [file 41598_2026_54258_MOESM1_ESM.docx]

# Supplement 1: Measures used in the study

PHQ-9

The Patient Health Questionnaire-9 (PHQ-9) has good psychometric properties and is widely used in primary care [1].. It focuses on assessing depression over the past two weeks using nine items on a scale from 0 "not at all" to 3 " nearly every day" with a total score ranging from 0 to 27. The severity of depression is categorized as minimal (0-4), mild (5-9), moderate (10-14), moderate severe (15-19) and severe (20-27) [1].

BSS

The Beck Scale for Suicide Ideation (BSS) asks about SI of the past week using 21 items on a three-point scale (0-2) with good psychometric properties [2]. Items 1-5 are used as a screening assessment. All subsequent items are answered if there is a suicidal wish (item 4) or a refusal to save oneself in a life-threatening situation (item 5). The total score ranges from 0 to 38, with a higher score indicating more severe suicidal ideation without classifying cut-offs [3].

BRFL

The Brief Reasons for Living (BRFL) [4] was factor analysed and validated and derived from the Reasons for Living [5]. It is a brief, 12-item measure to assess positive attitudes that support the desire to live. The inventory has six subscales rated on a 6-point scale, with (1) representing 'not at all important' and (6) representing 'extremely important': fear of suicide, family responsibility, survival and coping beliefs, childhood concerns, moral objections and fear of social disapproval [6]. This factor structure is the basis for the reasons listed in the last item SuPr-10.

GAD-7

The Generalised Anxiety Disorder-7 (GAD-7) consists of 7 questions about anxiety symptoms in the past 2 weeks and is a commonly used screening instrument for the severity of generalised anxiety disorder (GAD) [7]. Responses are rated on a 4-point scale from 0 "not at all" to 3 "almost every day". Severity is measured using the total score: Minimal or no anxiety (0-4), mild anxiety (5-9), moderate anxiety (10-14) and severe anxiety (15-21). It has been validated in primary care settings and shown to be valid and reliable [8].

PHQ-15

The Patient Health Questionnaire-15 (PHQ-15) is also part of the PHQ-D [9]. It consists of 15 items and assesses somatic symptoms over the past four weeks. Each item is scored from 0 ('not bothered at all') to 2 ('bothered a lot'), with a total score ranging from 0 to 30. The total score classifies somatic symptoms as minimal (0-4), low (5-9), moderate (10-14) and high (15-30) [10]. It has good psychometric properties for use in primary care [11].

PC-PTSD-5

The Primary Care PTSD Screen is a screening tool to identify patients with post-traumatic stress disorder according to DSM-5 criteria [12]. It consists of 5 items asking about PTSD symptoms in the past 4 weeks on a dichotomous yes/no scale. A score of 3 or more positive responses is an indicator of PTSD that should be followed by a more detailed assessment. The instrument is used for screening in primary care and has been validated in German [13].

Acceptability questionnaire

A modified acceptability questionnaire based on that used by Gräfe et al. for the evaluation of the PHQ-9 in 2004 [9] was also used for the evaluation of the new SuPr-X instrument. It focuses on assessing respondents' feedback on their experience of filling in a health questionnaire. The questionnaire contains five key items: Patient satisfaction, difficulty, time consumed, perceived usefulness for medical treatment, willingness to complete similar questionnaires in the future.

**References**

1. Kroenke, K., Spitzer, R.L. & Williams, J.B.W., *The PHQ-9.* Journal of General Internal Medicine, 2001. **16**(9): p. 606-613.DOI: 10.1046/j.1525-1497.2001.016009606.x.

2. Kliem, S. & Brähler, E., *Beck-Suizidgedanken-Skala (BSS)*. 2016.

3. Beck, A.T. & Steer, R.A., *BSI, Beck Scale for Suicide Ideation: Manual*. <https://books.google.de/books?id=bFFSHAAACAAJ1991>: Psychological Corporation.

4. Ivanoff, A., Jang, S.J., Smyth, N.J. & Linehan, M.M., *Fewer reasons for staying alive when you are thinking of killing yourself: The Brief Reasons for Living Inventory.* Journal of Psychopathology and Behavioral Assessment, 1994. **16**: p. 1-13.

5. Linehan, M.M., Goodstein, J.L., Nielsen, S.L. & Chiles, J.A., *Reasons for staying alive when you are thinking of killing yourself: the reasons for living inventory.* Journal of consulting and clinical psychology, 1983. **51**(2): p. 276.

6. Cwik, J.C., Siegmann, P., Willutzki, U., Nyhuis, P., Wolter, M., Forkmann, T., Glaesmer, H. & Teismann, T., *Brief reasons for living inventory: a psychometric investigation.* BMC psychiatry, 2017. **17**: p. 1-11.

7. Spitzer, R.L., Kroenke, K., Williams, J.B. & Löwe, B., *A brief measure for assessing generalized anxiety disorder: the GAD-7.* Arch Intern Med, 2006. **166**(10): p. 1092-7.DOI: 10.1001/archinte.166.10.1092.

8. Löwe, B., Müller, S., Brähler, E., Kroenke, K., Albani, C. & Decker, O., *GAD-7-Generalized Anxiety Disorder Screener-deutsche Fassung (PSYNDEX Tests Info).*

9. Gräfe, K., Zipfel, S., Herzog, W. & Löwe, B., *Screening psychischer Störungen mit dem“Gesundheitsfragebogen für Patienten (PHQ-D)“.* Diagnostica, 2004. **50**: p. 171-181.DOI: 10.1026/0012-1924.50.4.171.

10. Kroenke, K., Spitzer, R.L. & Williams, J.B., *The PHQ-15: validity of a new measure for evaluating the severity of somatic symptoms.* Psychosomatic medicine, 2002. **64**(2): p. 258-266.

11. van Ravesteijn, H., Wittkampf, K., Lucassen, P., van de Lisdonk, E., van den Hoogen, H., van Weert, H., Huijser, J., Schene, A., . . . Speckens, A., *Detecting somatoform disorders in primary care with the PHQ-15.* Ann Fam Med, 2009. **7**(3): p. 232-8.DOI: 10.1370/afm.985.

12. Prins, A., Bovin, M.J., Smolenski, D.J., Marx, B.P., Kimerling, R., Jenkins-Guarnieri, M.A., Kaloupek, D.G., Schnurr, P.P., . . . Tiet, Q.Q., *The Primary Care PTSD Screen for DSM-5 (PC-PTSD-5): Development and Evaluation Within a Veteran Primary Care Sample.* J Gen Intern Med, 2016. **31**(10): p. 1206-11.DOI: 10.1007/s11606-016-3703-5.

13. Schäfer, I. & Schulze, C., *Deutsche Version des “Primary Care Posttraumatic Stress Disorder screening questionnaire”.* Universität Hamburg, 2010.

# Supplement 2: Item analyses

Table a. Two-factor solution - loading matrix

|  | **Factors** | |  |
| --- | --- | --- | --- |
| **Items: In the past two weeks…** | **1** | **2** | |
| 1. …I was confident | **0.901** | -0.000 | |
| 1. …I had goals in life. | **0.590** | -0.035 | |
| 1. …I felt able to cope with life and its difficulties. | **0.710** | -0.084 | |
| 1. …I was satisfied with my life. | **0.716** | 0.022 | |
| 1. …I have had the wish to die. | -0.085 | **0.828** | |
| 1. …I have thought about taking my own life. | 0.013 | **0.969** | |
| 1. …I have thought about a specific suicide method. | -0.056 | **0.904** | |
| 1. …I have thought about a specific suicide plan. | -0.042 | **0.934** | |
| 1. …I have had the urge to realize my suicide plan. | 0.042 | **0.980** | |
| 1. …I have made preparations for the time after my suicide. | **0.307** | **0.926** | |
| 1. ...I have intentionally injured myself and / or put myself in   danger, risking death | -0.032 | **0.651** | |

Table b. Item analyses

|  | | | | | | | | | | | | |
| --- | --- | --- | --- | --- | --- | --- | --- | --- | --- | --- | --- | --- |
| F | **Item** | **M** | | **SD** | | **Skewness** | **Kurtosis** | **Item diff.** | **Item**  **discr.** | **α,** when item deleted | **Com.** |  |
| 1 | **1** | 1,24 | | 0,76 | | 0,19 | -0,31 | 0,41 | 0,72 | 0,73 | 0,81 |  |
|  | **2** | 1,41 | | 0,87 | | 0,02 | -0,70 | 0,47 | 0,57 | 0,81 | 0,35 |  |
|  | **3** | 1,58 | | 0,80 | | 0,33 | -0,43 | 0,35 | 0,64 | 0,77 | 0,51 |  |
|  | **4** | 0,95 | | 0,80 | | 0,45 | -0,43 | 0,32 | 0,63 | 0,77 | 0,51 |  |
|  | **M** |  | |  | |  |  | **0,39** | **0,64** |  | **0,55** |  |
|  |  |  | |  | |  |  |  |  |  |  |  |
| 2 | **5** | 0,88 | | 1,06 | | 0,79 | -0,77 | 0,29 | 0,75 | 0,88 | 0,69 |  |
|  | **6** | 0,64 | | 1,00 | | 1,29 | 0,24 | 0,21 | 0,86 | 0,86 | 0,94 |  |
|  | **7** | 0,58 | | 0,95 | | 1,42 | 0,68 | 0,19 | 0,84 | 0,87 | 0,82 |  |
|  | **8** | 0,32 | | 0,72 | | 2,41 | 5,05 | 0,11 | 0,83 | 0,87 | 0,87 |  |
|  | **9** | 0,26 | | 0,66 | | 2,84 | 7,80 | 0,09 | 0,79 | 0,88 | 0,96 |  |
|  | **10** | 0,14 | | 0,50 | | 3,91 | 15,76 | 0,05 | 0,53 | 0,90 | 0,95 |  |
|  | **11** | 0,19 | | 0,55 | | 3,27 | 10,73 | 0,06 | 0,45 | 0,91 | 0,42 |  |
|  | **M** |  |  | |  | |  | **0,14** | **0,72** |  | **0,81** |  |
| TOTAL |  |  |  | |  | | \|  \|  \|  \|  \| \| --- \| --- \| --- \| --- \| | **0.23** | **0.69** |  | **0.71** |  |

Table c. Diagnostic Accuracy of SuPr-10 risk scale (Item 5.-9)

| Cut-Off | Sensitivity | Specificity | Youden-  Index | PPV  depressive symptoms* | NPV  depressive symptoms* |
| --- | --- | --- | --- | --- | --- |
| **≥1** | 83,17% | 56,17% | 39,34% | 34,20% | 92,42% |
| **≥2** | 78,22% | 68,77% | 46,98% | 40,68% | 92,02% |
| **≥3** | 66,34% | 74,58% | 40,91% | 41,68% | 89,00% |
| **≥4** | 59,41% | 79,90% | 39,31% | 44,74% | 87,79% |
| **≥5** | 51,49% | 82,81% | 34,29% | 45,06% | 86,17% |
| **≥6** | 45,54% | 87,65% | 33,20% | 50,25% | 85,46% |
| **≥7** | 38,61% | 89,59% | 28,20% | 50,39% | 84,20% |
| **≥8** | 33,66% | 92,25% | 25,92% | 54,34% | 83,55% |
| **≥9** | 28,71% | 94,67% | 23,39% | 59,62% | 82,90% |
| **≥10** | 25,74% | 95,88% | 21,63% | 63,14% | 82,50% |
| **≥11** | 20,79% | 96,85% | 17,64% | 64,40% | 81,70% |
| **≥12** | 18,81% | 98,06% | 16,87% | 72,68% | 81,52% |
| **≥13** | 14,85% | 98,06% | 12,91% | 67,74% | 80,79% |
| **≥14** | 7,92% | 98,55% | 6,47% | 59,89% | 79,62% |
| **≥15** | 2,97% | 98,55% | 1,52% | 35,90% | 78,76% |

Adapted from [36], p.80; A-priori probability suicide attempt: 21,5% in study sample with depressive symptoms.


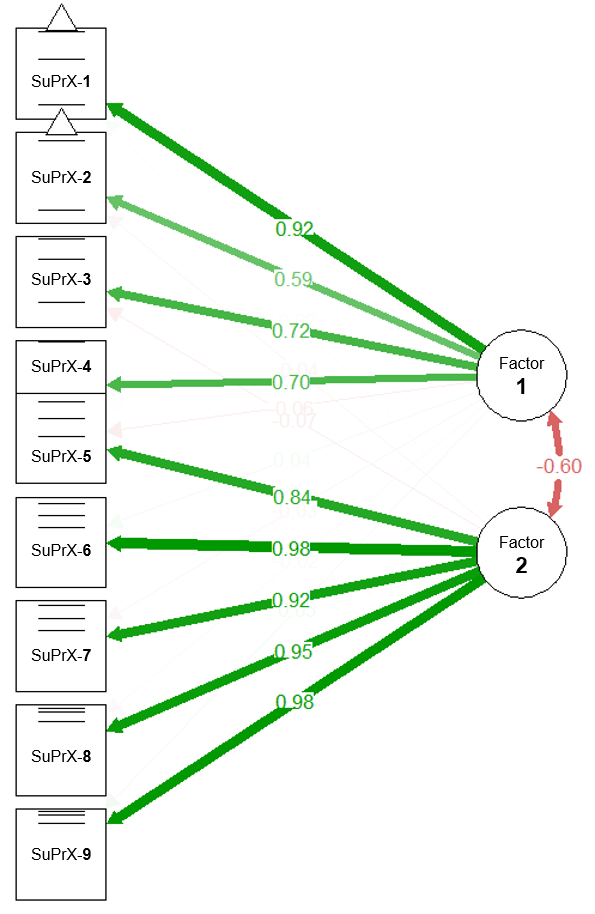


Figure a. SEM Plot of two factor solution with remaining SuPr-X items 1-9.

# Supplement 3 SuPr-10 final questionnaire

| SuPr-10 questionnaire  *Please read the questions carefully. There are no right or wrong answers. It is also okay if you cannot or do not want to give an answer. In this case, please write "n/a" next to it.* | | | | |
| --- | --- | --- | --- | --- |
| 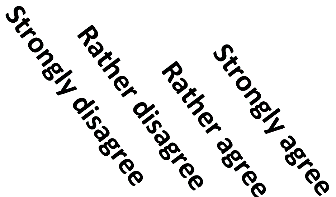  Part I: How strongly do the following statements apply to you? | | | | |
| 1. In the last two weeks, I was generally confident. | 0 | 1 | 2 | 3 |
| 1. In the last two weeks, I have had goals in life. | 0 | 1 | 2 | 3 |
| 1. In the last two weeks, I felt able to cope with life and its difficulties. | 0 | 1 | 2 | 3 |
| 1. In the last two weeks, I was satisfied with my life. | 0 | 1 | 2 | 3 |
| Total 1st-4th:  Part II: | | | | |
| 1. Sometimes people are feeling so depressed that they wish they were not alive. Have you ever wished you were not alive?  - No - Yes, if yes, when was the last time? ___________________________________________ | | | | |
| 1. Have you ever tried to take your own life?  - No - Yes If yes, when? _________________________  how often? __________________________   how? _______________________________ | | | | |
|  | | | | |
| *If you have suicidal thoughts, contact your doctor / therapist*  *or call the free telephone counseling service on 0800 1110111 (available 24 hours a day).* | | | | |

| Part III: How strongly do the following statements apply to you?  **Suicide means ending one‘s own life.* | 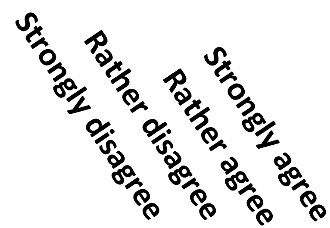 | |  |  |  |
| --- | --- | --- | --- | --- | --- |
| 1. In the last two weeks, I have wished to be dead. | | 0 | 1 | 2 | 3 |
| 1. In the last two weeks, I have thought about taking my own life. | | 0 | 1 | 2 | 3 |
| 1. In the last two weeks, I have thought about a specific suicide method*. | | 0 | 1 | 2 | 3 |
| 1. In the last two weeks, I have thought about a specific suicide plan*. | | 0 | 1 | 2 | 3 |
| 1. In the last two weeks, I have had the urge to realize my suicide plan*. | | 0 | 1 | 2 | 3 |
| Total 5th-9th:   1. Have you attempted suicide* in the last two weeks?  - Yes | | | | | |
| - No  🡪 10.1 What reasons prevented you from a suicide attempt?   *(multiple choice possible)* - Confidence in your own strength that you will be able to overcome the difficulties somehow - Faith or hope for improvement - Responsibility for others - Support from family, friends or other social contacts - The fear of death or suicide - Moral or religious concerns - Concern about disapproval or the reaction of the environment - Other reasons: _______________________________________ | | | | | |
| I would like to make the following additional comment: _____________________________________________________________________________________________________________________________________________________________________________________________________________________ | | | | | |

| SuPr-10 Fragebogen  *Bitte lesen Sie die Fragen sorgfältig durch. Es gibt keine richtigen oder falschen Antworten. Es ist auch in Ordnung, wenn Sie keine Antwort geben können oder wollen. Notieren Sie in diesem Fall bitte „k.A.“ daneben.* | | | | |
| --- | --- | --- | --- | --- |
| 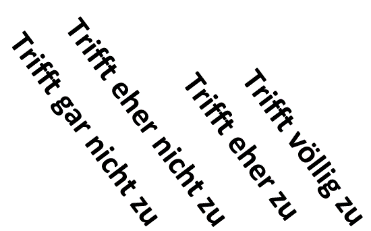  Teil I: Wie stark treffen folgende Aussagen auf Sie zu? | | | | |
| 1. In den letzten zwei Wochen war ich im Allgemeinen zuversichtlich. | 0 | 1 | 2 | 3 |
| 1. In den letzten zwei Wochen hatte ich Ziele im Leben. | 0 | 1 | 2 | 3 |
| 1. In den letzten zwei Wochen fühlte ich mich dem Leben und seinen Schwierigkeiten gewachsen. | 0 | 1 | 2 | 3 |
| 1. In den letzten zwei Wochen war ich alles in allem zufrieden mit meinem Leben. | 0 | 1 | 2 | 3 |
| Summe 1.-4.:  Teil II: | | | | |
| 1. Manchmal sind Menschen so sehr niedergeschlagen, dass sie den Wunsch haben, nicht am Leben zu sein. Haben Sie sich jemals gewünscht, lieber nicht am Leben zu sein?  - Nein - Ja wenn ja, wann zuletzt? ___________________________________________ | | | | |
| 1. Haben Sie jemals versucht sich das Leben zu nehmen?  - Nein - Ja wenn ja, wann? ________________________   wie oft? ______________________________  wie? _________________________________ | | | | |
|  | | | | |
| *Wenn Sie Suizidgedanken haben, wenden Sie sich an Ihre/n Behandler*in*  *oder an die kostenlose Telefon Seelsorge unter 0800 1110111 (täglich 24h erreichbar).* | | | | |

| Teil III: Wie stark trafen folgende Aussagen auf Sie zu?  **Suizid ist ein anderes Wort für Selbsttötung.* | 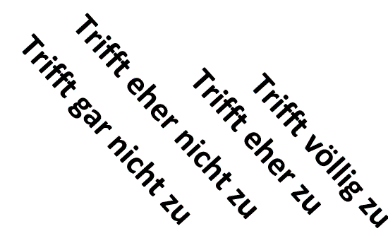 | |  |  |  |
| --- | --- | --- | --- | --- | --- |
| 1. In den letzten zwei Wochen hatte ich den Wunsch zu sterben. | | 0 | 1 | 2 | 3 |
| 1. In den letzten zwei Wochen habe ich darüber nachgedacht mir das Leben zu nehmen. | | 0 | 1 | 2 | 3 |
| 1. In den letzten zwei Wochen habe ich mir Gedanken zu einer bestimmten Suizidmethode* gemacht. | | 0 | 1 | 2 | 3 |
| 1. In den letzten zwei Wochen habe ich über einen konkreten Suizidplan* nachgedacht. | | 0 | 1 | 2 | 3 |
| 1. In den letzten zwei Wochen hatte ich den Drang meinen Suizidplan* umzusetzen. | | 0 | 1 | 2 | 3 |
| Summe 5.-9.:   1. Haben Sie in den letzten zwei Wochen einen Suizidversuch* unternommen?  - Ja | | | | | |
| - Nein  🡪 10.1 Welche Gründe schützten Sie vor einem Suizidversuch?   *(Mehrfachauswahl möglich)* - Das Vertrauen in die eigene Stärke, die Schwierigkeiten schon irgendwie bewältigen zu können - Die Zuversicht oder Hoffnung auf Besserung - Die Verantwortung für andere - Die Unterstützung durch Familie, Freunde oder andere soziale Kontakte - Die Angst vor dem Tod oder der Selbsttötung - Moralische oder religiöse Bedenken - Die Sorge vor Missbilligung oder der Reaktion des Umfelds - Sonstige Gründe: _______________________________________ | | | | | |
| Ich möchte noch folgende weitere Anmerkung machen: _____________________________________________________________________________________________________________________________________________________________________________________________________________________ | | | | | |

# Supplement 4 - Differences in preventive reasons across treatment settings

*GP= General practitioner, PT= Psychotherapist, PSY= Psychiatrist (outpatient), DCP= Dayclinic patients, NASIP = non-acutely suicidal inpatient, ASIP= acutely suicidal inpatient*

# Supplement 5 Worksheets and handouts

**Handout: Recommended Application of the SuPr-10 (Risk and Protective Scales)**

*Primary-care workflow for interpreting SuPr-10 and translating scores into immediate clinical actions.*

**1) Using the SuPr-10 Risk Scale (sum of items 5–9)**

**Elevated risk (any score > 0)**

- Treat any non-zero risk score as elevated risk; increase vigilance and co-create a brief safety plan.
- Use the patient’s stated reasons for not attempting (elicited in SuPr-10) as anchors in the plan.
- Prioritize interventions that strengthen self-efficacy and mobilize social support (see Crisis Management Handout).

**Consider inpatient evaluation (≥ 4 points)**

- Discuss the option of inpatient evaluation openly; document clinical reasoning.
- Arrange rapid follow-up and ensure a written safety plan is in place.

**Very high concern (≥ 7 points)**

- Score patterns comparable to acutely suicidal inpatients; ensure very close monitoring, ideally in inpatient care.
- If inpatient care is not immediately feasible, organize same-day psychiatric assessment and continuous observation until handover.

**Important**

- Questionnaires complement but do not replace clinical judgment; do not make decisions based on scores alone.

**2) Interpreting the SuPr-10 Protective Scale (items 1–4)**

*The protective scale contextualizes resources and current well-being; it should* ***not*** *be used as a stand-alone risk indicator.*

- Protective ≤ 5: best combined accuracy for indicating current depression/suicidality → consider closer follow-up.
- Protective ≤ 4: best accuracy for indicating a history of attempts; review suicide-/psychiatric history, social supports, coping strategies, and help-seeking; may indicate increased risk.
- Protective ≤ 2: resembles the profile of acutely suicidal inpatients → intensify monitoring; ensure rapid reassessment.
- Reminder: a **low** protective score alone does **not** indicate high suicide risk; a **high** protective score does **not** rule it out.
- Repeat risk assessment when the protective score is very low, even if all risk items are negated.

**Handout: Crisis Management (Acute Help & Self-Management)**

*A brief guide for primary care: first steps in crisis intervention and strengthening self-management.*

**1) Create a safe environment**

- Identify personal triggers/situations that intensify distress or suicidal thoughts.
- Review access to potentially lethal means (medications, sharp objects, high places, etc.) and plan to limit access or remove items.
- Agree on concrete steps to reduce acute risk at home (who will help, when, and how).

**2) Immediate coping strategies (right now)**

- Ask yourself: “How do I want to feel ten minutes from now?” Choose one short action that moves you in that direction.
- Write down your thoughts
- Breathing: slow, paced breathing (e.g., in 4 seconds, out 6–8 seconds) for 2–3 minutes.
- Body: brief movement, cold water on wrists/face, muscle squeeze-and-release cycles.
- Attention shift: name 5 things you can see/hear/feel around you
- Go for a walk
- Scream into a pillow
- Listen to loud music
- Safe places: identify places where you feel safer; go there if possible.
- Contact: call or message a trusted person; agree on brief check-in calls if needed.
- Short decision: decide what you will do in the next 10 minutes and then for the next hour.

**3) Psycho-hygiene to promote well-being**

- Daily structure with breaks.
- Regular movement.
- Regular, balanced meals and sufficient fluids.
- Adequate, regular sleep routine.
- Attend indicated medical care; avoid alcohol/drugs as coping.
- Digital hygiene: limit doom-scrolling; schedule device-free times.
- Nature & pleasurable activities: brief doses every day.

**4) Retrieve knowledge and check self-management**

- After creating a safety plan, rehearse how to use it: “Suppose you wake up tomorrow and feel very bad; what will you do?”
- Write down the plan and keep it accessible (wallet/phone).
- Digital options: safety-plan apps (e.g., LifeStep, KrisenKompass).

**Worksheet: My Crisis Plan**

**Self-care checklist**

- Balanced daily structure with breaks.
- Enough physical activity.
- Regular, balanced meals.
- Enough to drink.
- Regular and sufficient sleep.
- Attend medical appointments and take medications as indicated.
- No (excessive) use of alcohol, nicotine, medications, or drugs.

**My early warning signs of psychological distress**

**Skills that help me quickly**

- Breathing / body / attention shift.
- Short grounding routines.
- Brief movement or going outside.
- Contacting a trusted person.
- Going to a safe place.

**Safe places / safe activities**

**Trusted contacts (names & phone numbers)**

**Emergency numbers (local/national)**

- Emergency medical services (ambulance): **112**
- On-call medical service: **116 117**
- Crisis hotline / lifeline: **0800 111 0 111**
- Poison control Germany: **030-19240**

**Blank Emergency Telephone List (Template)**

*Please fill in local services and trusted contacts. Keep a printed copy near your phone and save numbers on your mobile.*

- Psychiatric crisis service: ____________________________
- Crisis helpline / lifeline: ____________________________
- On-call medical service: ____________________________
- Emergency (ambulance/fire): ____________________________
- Poison control: ____________________________
- Local hospital / psychiatric emergency department: ____________________________
- Primary care practice: ____________________________
- Trusted contact 1 (name/phone): ____________________________
- Trusted contact 2 (name/phone): ____________________________

# **Clinical Quick Guide: Interpreting and Applying the SuPr-10 in Primary Care**

| **SuPr-10 Risk Scale (items 5–9)** | **Clinical Interpretation** | **Recommended Clinical Actions** |
| --- | --- | --- |
| **> 0** Elevated Risk | Any non-zero score indicates presence of suicidal ideation or impulses. | • Treat as elevated risk. • Increase vigilance and co-create a short safety plan. • Use patient’s stated reasons for not attempting (item 10.1) as anchors for safety planning. • Address modifiable stressors; mobilize supports. |
| **≥ 4** High Concern | Risk pattern suggests significant suicidal ideation or planning. | • Discuss potential inpatient evaluation. • Document reasoning and patient preference. • Arrange rapid follow-up and ensure written safety plan. |
| **≥ 7** Very High Concern | Profile comparable to acutely suicidal inpatients. | • Ensure close monitoring and rapid psychiatric assessment. • If inpatient care is not immediately possible, ensure same-day crisis contact and continuous observation until handover. |
| **General note** | Questionnaires complement but do not replace clinical judgment. | • Always discuss results directly with the patient. • Base near-term decisions primarily on risk scale and clinical impression. |

**Note for clinicians:**
Protective factors are intended to **facilitate conversation and safety planning**, not to offset risk. The SuPr-10 is designed as an **adjunctive screening and decision-support tool**, to be used **alongside the PHQ-9 and clinical judgment**.
